# Real-Time Automatic Calculation of Euro Coins and Banknotes in a Cash Drawer

**DOI:** 10.3390/s19112623

**Published:** 2019-06-09

**Authors:** Manuel Cereijido, Fernando Nuño, Alberto M. Pernía, Miguel J. Prieto, Pedro J. Villegas

**Affiliations:** Área de Tecnología Electrónica, Universidad de Oviedo, Edificio Departamental No. 3. Campus Universitario, 33203 Gijón, Spain; manuelcereijido@gmail.com (M.C.); amartinp@uniovi.es (A.M.P.); mike@uniovi.es (M.J.P.); pedroj@uniovi.es (P.J.V.)

**Keywords:** bluetooth, capacitance, cash-register, currency-counting, embedded, Euro, load cell, low-cost, money, real-time, smartphone

## Abstract

A very interesting and useful complement to classical cash-registers is presented in this paper, coming up with a real-time auto-counting solution for the money inside a cash drawer. The system allows knowing not only the total amount of money but also how many coins and banknotes there are of each value. The embedded solution developed has been intended to become a low-cost solution, allowing better control over the money and helping both owners and workers in the establishments. By using this system, new utilities including automatic final balancing, instant error handling when making operations, and the lack of certain types of banknotes or coins inside the drawer or the excess of some in a certain compartment, could be implemented. Coins-counting solution is based on their weight, and small individual scales made by load cells have been integrated in each coin compartment. With respect to the banknotes, an innovative alternative based on the electrical properties of capacitors is presented. Additionally, considering the relevance of interoperability in today’s systems, a Bluetooth module has been integrated into the system, allowing for data to be accessed remotely from any smartphone, tablet or computer within the range of the module. In this work, an Android application to both control and interact with the system has also been designed.

## 1. Introduction

The invention of the cash register, dated around the end of the 19th century, responded to the need for quicker and more accurate ways of providing summaries of daily transactions at points of sale [1]. Mechanical cash registers improved continuously during the following years, but it was not until the decade of 1950s and 1960s when the introduction of electronic cash registers took place.

During the last decades, the improvements added to cash registers have not been significant. Nevertheless, new products intended to improve and facilitate the use and control of money have appeared, as is the case of the currency-counting machine [2]. The continued development of technology enables the emergence of new products as well as the enhancement of those already in the market. Thus, it is possible to find works related to optimization of the use of cash registers in malls [3] or patents focused on identifying the cashier, the customer, the type of operation, etc. [4,5]. There are also many papers in the literature dealing with currency recognition using machine vision [6,7,8,9], but they all need the coins or banknotes to be distinctly placed on a surface suitable for the camera to detect them. Some of these recognition systems can operate properly if the coins/banknotes are moving on a conveyor, but that is not what happens in stores and supermarkets. Other works describe systems that succeed in identifying the currency going through some kind of slot [10,11], which also allows for accurate accounting of this money.

There are not, however, so many works related to counting the money once it is inside the drawers of such cash register machines. This is important not only in terms of accounting (so as to know the amount of money available in the cash machines at any given time), but also in order to guarantee that shortages in cash registers are avoided. This is a problem that has always concerned the owners of this type of business, but so far solutions have mostly come in terms of organization strategies [12,13]; not many technological solutions (if any) have been proposed to address this issue.

This paper aims to present the development of a prototype for a new product capable of counting the exact amount of money inside the cash drawer in real-time, thus combining features of both cash registers and currency-counting machines. Depending on the accuracy of the system designed, its functionality will allow it to carry out accounting tasks or be limited to warn about possible shortage situations in the cash registers.

Also, the rise in the use of wireless communication systems, together with the growing interest in low-power and portable devices, have been taken into consideration during the design of the prototype. Even though products with similar characteristics can already be found in the market [14], they are bulky and expensive (mainly focused on large supermarket chains or alike). In this sense, the prototype proposed in this paper also aims to prove the feasibility of a manageable and low-cost solution, which would increase its scope of application.

With regards to the distribution of the money within the cash drawer and based on today’s commercial products, it has been chosen to divide the cash drawer into 12 different compartments: one for each euro coin denomination (eight in total) and the other four intended for 5€, 10€, 20€ and 50€ banknotes. In any case, an extension to more compartments could be added in a simple way.

According to this, the major contributions to be conveyed by this work can be summarized as follows:

- Development of a novel capacitive sensor meant to count banknotes in a cash drawer.
- Integration of the developed sensor with other well-known solutions to count coins and widely-used wireless communication systems in order to produce a joint cash-count system.
- Contribution to avoiding the problem of shortages in cash registers. Possibility to develop a real-time accounting system if enough accuracy is obtained.
- Validation of the performance of the capacitive sensor developed by means of tests that confirm the accuracy and repeatability of the measurement.

## 2. System Overview

As it has been mentioned in the previous section, the aim of this work is to develop a solution for counting both coins and banknotes in real-time. Therefore, it is essential to find a physical property and, consequently, a sensor that allows different coins and banknotes to be identified.

The most noteworthy physical aspects of coins are their diameter, thickness, and weight. Since the Euro has been chosen as the reference, it can be seen from Table 1 that the diameter and weight are different for each coin. Considering that prior actions like sorting would be necessary in the case of selecting the diameter as the differentiating feature, the weight has resulted to be the most suitable option.

The viability of the commercial solutions proposed this far regarding the counting of banknotes [15] is limited to some extent. That is why instead of trying to identify the number of banknotes by means of a direct measurement of a physical feature such as their size or colour [16] as indicated in Table 2, it has been decided that banknotes be used as a means to affect or influence an auxiliary, measurable property. In this way, an alternative to the previous methods is unveiled and will be further analysed in Section 4.

Any measurement taken from the real world and intended for further usage into a digital system must be processed to some extent. This process is known as Data Acquisition (DAQ) and it comprises all steps from the measurement of the physical phenomenon to the processing of these data by the system, e.g., a computer or a microcontroller. The details of the DAQ processes needed to obtain the data from the sensors are included in Section 3 and Section 4.

Nevertheless, it does not matter whether the data from the real world can either be obtained or processed if they cannot be accessed afterwards. That is the reason communications play a key role in most of the systems. Taking into consideration the main objectives of the prototype, a wireless communication and, more precisely Bluetooth technology, has been chosen as the preferred option. The absence of cables increases the manageability of the product and simplifies its mechanical design. Additionally, using Bluetooth technology eliminates the need to acquire a specific piece of equipment to receive the information provided by the system, for most secondary devices such as smartphones, tablets or computers include Bluetooth modules that allow for interoperability with the prototype designed [17].

At this point, the fundamental pillars of the prototype and the technologies that will be used in the design stage have been analysed and defined respectively.

## 3. Coins Calculation System

As previously mentioned, the solution proposed in this paper uses the weight as the differentiating feature between coins. There are already commercial products that use this method to count the number of coins or other items [18]. Due to the existing direct proportionality between force and mass, a force sensor has been chosen to measure the weight.

Among all different kinds of force sensors, strain gauges’ load cells are the most widely used in the implementation of small, high-precision scales. The beam or structural element of the load cell needs to be selected depending on the force the load cell will be submitted to. In the case of a scale, the forces acting on the load cell are unidirectional and therefore, a single-bending beam load cell is enough.

Since each coin denomination has a different weight, it might seem that a single load cell would be enough to count all the money. However, this solution has many drawbacks, including the fact that it would be impossible to calculate the initial amount of money after rebooting the system or checking whether a coin has been placed in its corresponding compartment, among others. Therefore, it has been decided that one load cell sensor CZS639M [19] be used for each coin compartment, eight in total. Even though both the electrical and mechanical design of the prototype becomes more challenging like this, the capabilities of the system increase.

**Figure 1:**
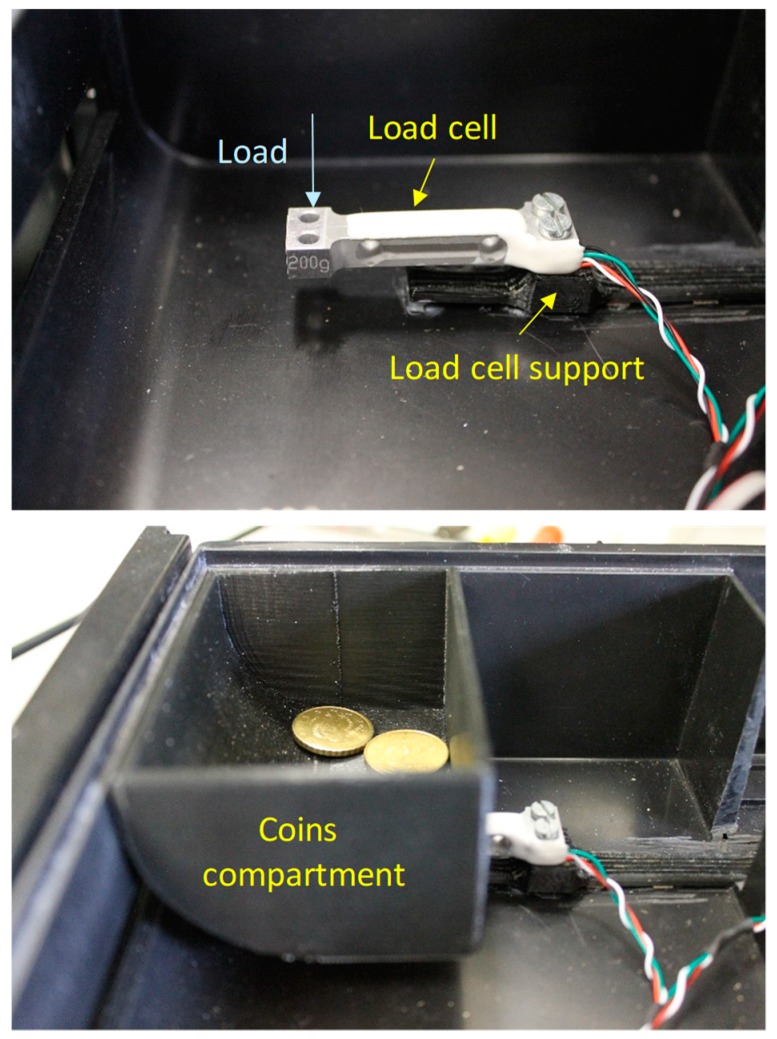
Load cell mount (**top**) and coins compartment assembly (**bottom**).

Figure 1 shows the detail of the assembly of each of the load cells in the drawer (top picture); the fixed end is fastened to a support, while the free end is cantilevered to allow downward deflection as the load is applied. The bottom picture (Figure 1) shows the coin compartment fitted on the load cell; in this way, the compartment (weight is 43 g) and the coins inside act vertically downwards. The load cell support and the coin compartment have been custom-designed and manufactured using a 3D printer. More details about mechanical design are included in Section 5 of this paper.

**Figure 2:**
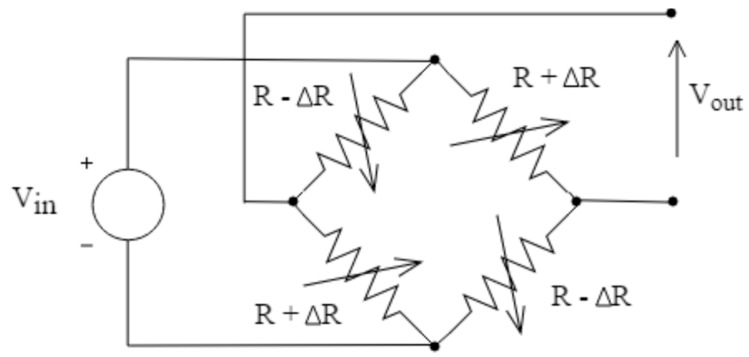
Four-point bridge sensor.

The variation of the electrical resistance in the strain gauges can be measured by means of a Wheatstone bridge. In those cases where it is possible to have strain gauges subjected to both tension and compression due to symmetry, as it is the case in the proposed system, the configuration shown in Figure 2 is the most suitable one. The output voltage of this setup, expressed by (1), follows a linear evolution with respect to the applied force and its sensitivity is up to four times higher in comparison with other configurations.

*V*_out_ = (Δ*R*/*R*)·*V*_in_ (1)

This solution to determine the number of coins in a separate compartment (using a strain gauge and a Wheatstone bridge to weigh them) is definitely not unknown to researchers in the field. In fact, this is but an implementation of an invention protected by US patent number 5,756,977 [20]; an implementation of this idea has also been protected by means of US patent number 10,127,776 [21]. Yet, it has been included in the paper so as to present the overall integration of the system, which uses different approaches to reach the final goal of the prototype as a whole.

It is usually far more challenging (and not at all mentioned in the patent indicated above) to amplify the typically low signals to values that can be easily handled by the processing system. In this case, the main characteristics of the selected load cell for our prototype are: rated load 200 g; sensitivity 0.6 mV/V; supply voltage 5 V to 10 V and nonlinearity ± 0.03%F.S. The magnitude of the bridge output voltage, as expected, does not exceed a few millivolts and, therefore, a high amplification gain needs to be added in the DAQ process.

The differential nature of the output signal coming from the bridge makes it necessary to use a differential input amplifier. Given the need for high-precision resistors and the reduced dimensions of the setup, an instrumentation amplifier is the most suitable choice for the goal of the project.

**Figure 3:**
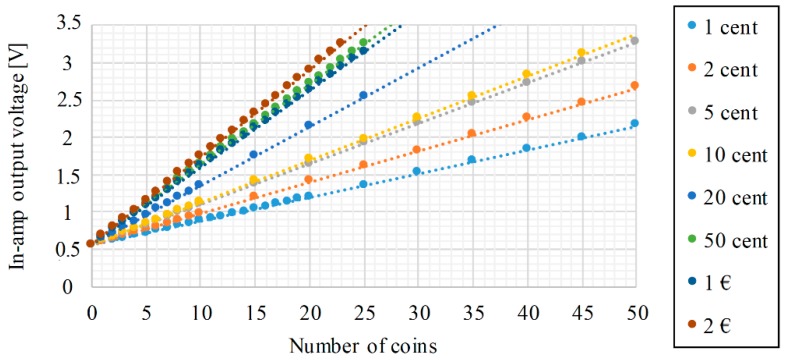
Graphical representation of the in-amp output voltage as a function of weight.

The instrumentation amplifier selected for the development of the project is the AD627 from Analog Devices [22]. Especially designed for transducer interfacing, its micropower consumption makes it suitable for portable battery-powered instruments and is capable of providing large gains without the need to include additional filtering stages. The selection of the gain depends on the input and output voltage ranges required for the application. In this case, it has been decided to adjust the gain so that a load of 200 g does not exceed the maximum reference voltage for the Analog to Digital Converter (3.3 V). One of the remarkable features of the AD627 is the possibility to adjust the gain by changing the value of only one external resistor. A 935 gain has been finally chosen and the performance of the setup is summarized in Figure 3. The presence of a non-zero voltage value without coins (0.5 V) is due to the weight of the compartment.

The voltage at the output of the instrumentation amplifier is connected to a microcontroller analog input. Since these devices are only capable of handling digital data, information contained in analog signals requires a previous conversion. Even though there are individual integrated circuits intended only to implement such an analog-to-digital conversion, the process is so common that most of the microcontrollers include an Analog to Digital Converter (ADC) module. In the development of this solution, the 12-bits ADC module integrated in the microcontroller chosen has been used, thus reducing the complexity of the system.

## 4. Banknotes Calculation System

There already exist solutions capable of counting the exact amount of money in banknotes, but they are quite bulky and slow [23]. US patent number 5,756,977 mentioned earlier [20] suggests that banknotes in cash drawers can also be counted from their weight; however, very sensitive load cells are needed in this case and the presence of used bills can cause significant errors. The patent itself states that *“although procedures for counting them by weighing which are accurate have been developed, (…) this may be at the cost of a higher cost of the system.”*

Thus, an alternative method, other than weighing them, is proposed in this paper to identify the number of banknotes present in the cash drawer. The key factor of this alternative method has to do with the properties the material banknotes are made of, pure cotton-fibre, which in turn results in being a dielectric material. The basic idea consists in placing the banknotes between two conductive plates; a change in the quantity of banknotes will lead to a variation of the capacitance between these two plates. Since all materials have a relative permeability or dielectric constant greater than unity, the capacitance will always be increased by inserting a dielectric.

ε = ε_0_ · ε_r_ (2)

where:
ε absolute permittivity of free space (F/m)ε_0_ permittivity of free space (F/m)ε_r_ relative permittivity (1)

Even though the relative permittivity of the dielectric material depends on many factors including temperature, pressure and frequency, the application proposed in this report does not imply significant variations in those properties. Thus, by keeping the surface of the parallel plates constant, the capacitance of the capacitor depends on the distance between the plates only (3) and, therefore, on the amount of banknotes between them.

*C* = (*A* · ε)/*d* (3)

where:
*C* capacitance (F)*A* surface of the plates (m^2^)ε absolute permittivity (F/m)*d* distance between plates (m)

In a cash drawer, there is a little tray for each kind of banknote. In each of these compartments, there is a spring and a metal clip that presses the banknotes together. In our case, a new mechanical design adapted to the existing metal clip and spring has been developed. In order to do so, a new bill holder has been designed and manufactured using a 3D printer (see Section 5, Section 5.4). This new element can hold the top conductive plate of the parallel plate capacitor while guaranteeing no electrical contact with the metal clip. Note that direct contact between the metal clip and the top conductive plate must be avoided, since both elements are conductive and such a contact might affect the expected value of capacitance. Physical implementation of the other conductive plate (that opposite to the one mounted on the bill holder) is achieved by gluing it directly on the cash drawer, since the bottom of the cash drawer is completely flat and made of non-conductive materials.

**Figure 4:**
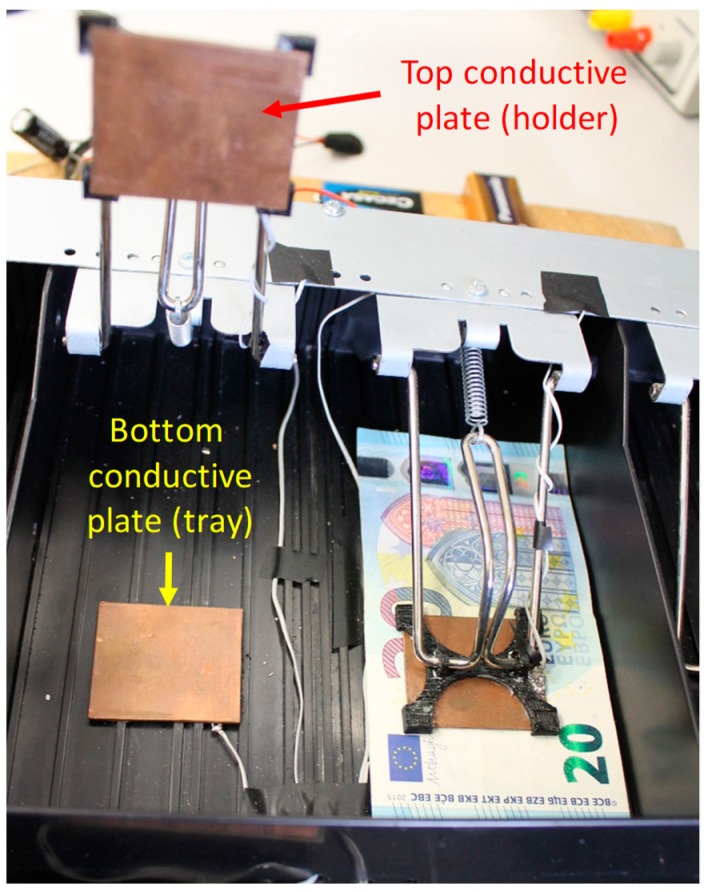
Adapted compartment without banknotes (**left**) and with 20 euro banknotes (**right**).

Figure 4 shows the arrangement of the two conductive plates and the new bill holder designed.

**Figure 5:**
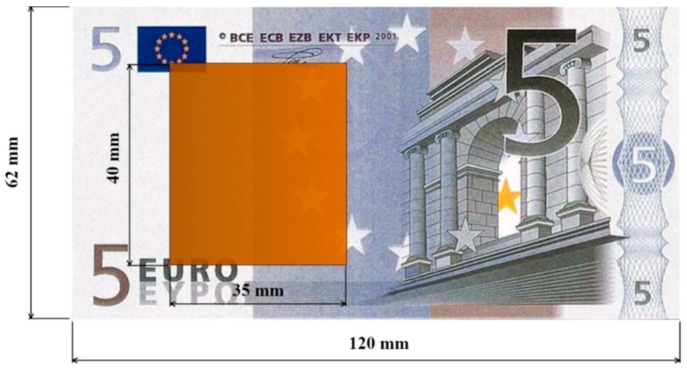
Dimensions of the conductive plates compared to a 5€ banknote.

To ensure that every Euro banknote fills the space between the plates regardless of its position in the compartment and to facilitate the introduction and withdrawal of the bills, the conductive plates were built 40 mm in width and 35 mm in length (Figure 5). As far as the material used for these plates is concerned, 1-mm-thick copper foil was used due to its being readily available.

**Figure 6:**
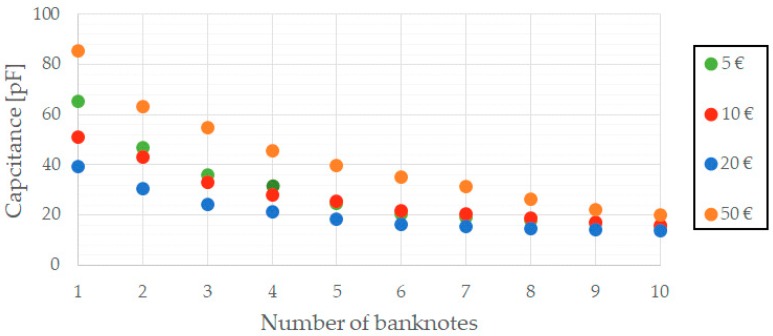
Evolution of the capacitance depending on the banknote value.

With the aid of an impedance analyser, the capacitance of the capacitor formed by the two parallel copper plates was measured for different number of bills. The results obtained are shown in Figure 6. Further analysis of the data is included in Section 7. The system to develop must be able to identify these capacitance variations and, hence, derive the number of banknotes inside the compartment.

Some of the simplest capacitance meters are based on the time it takes to charge a capacitor. Assuming the capacitor is included in an RC circuit where the value of *R* is known, it is possible to calculate the capacitance out of the charging time of the capacitor. This method, though simple, holds several inaccuracies related to knowing the exact value of the resistor or precisely determining the final voltage across the capacitor.

**Figure 7:**
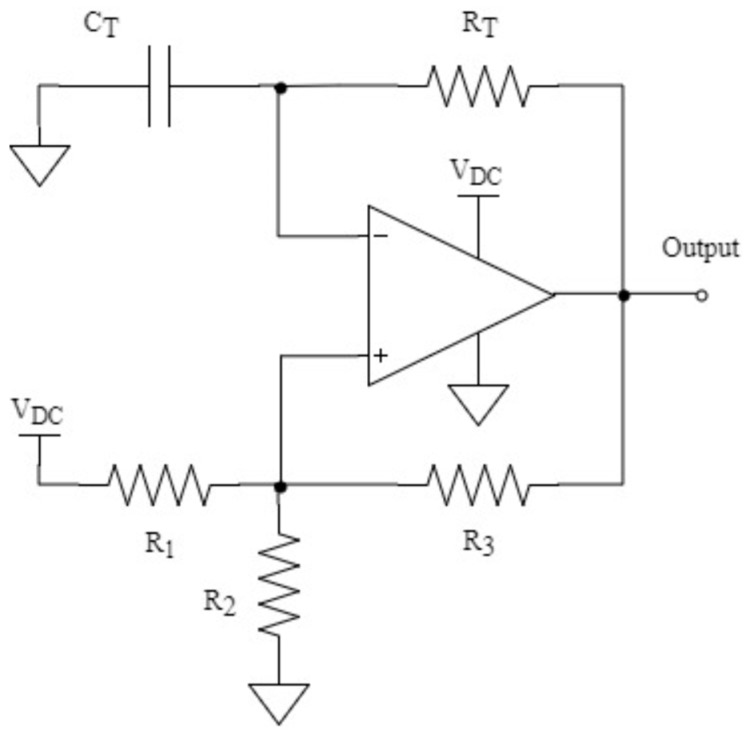
Multi-vibrator circuit (CT depends on the amount of banknotes between plates).

Aiming to increase the robustness and reliability of the system, an alternative based on an oscillatory circuit has been considered in this paper. By measuring the frequency of the signal produced in such a circuit, it is possible to determine the value of the capacitor included in it. There are several topologies that can provide an oscillatory output like the one needed; Figure 7 shows the one used in the design presented in this paper.

The design of the topology in Figure 7 is a typical application of the MCP6543 comparator from a Microchip, optimized for low-power applications. It should be noticed that this integrated circuit includes an enable pin (Chip Select), thus allowing for an even larger reduction of its consumption.

To design a multi-vibrator such as the one in Figure 7, the first step is to choose the value of the resistors *R*_1_, *R*_2_ and *R*_3_, which form the hysteresis feedback path. These resistors are closely related to the threshold voltages, *V_TH_* and *V_TL_*, whose values must be evenly spaced within the common mode range of the comparator and centered on *V*_DC_/2.

**Figure 8:**
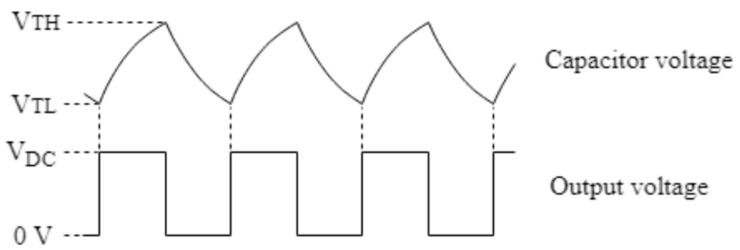
Waveforms at the non-inverting input and at the output of the comparator.

To limit the power dissipation in *R*_1_ and *R*_2_, the continuous current flowing through them must be reduced and thus, their value should be greater than 1 kΩ. In addition to that, large values of *R*_3_ (100 kΩ–10 MΩ) can produce voltage offsets at the non-inverting input due to the comparator’s input bias current. For the above reasons, it has been decided to use 10 kΩ resistors in the hysteresis feedback path. The remaining components of the topology, *R_T_* and *C_T_*, form a time delay network between the output and the inverting input, which consequently determines the oscillation frequency. Figure 8 shows the most important waveforms obtained in the circuit of Figure 7.

In the end, the mathematical expression that relates the oscillation frequency of the output, *F*_osc_, and the capacitance, CT, is given by (4).

(4) Fosc=12·RT·CT·ln(VTHVTL)

**Figure 9:**
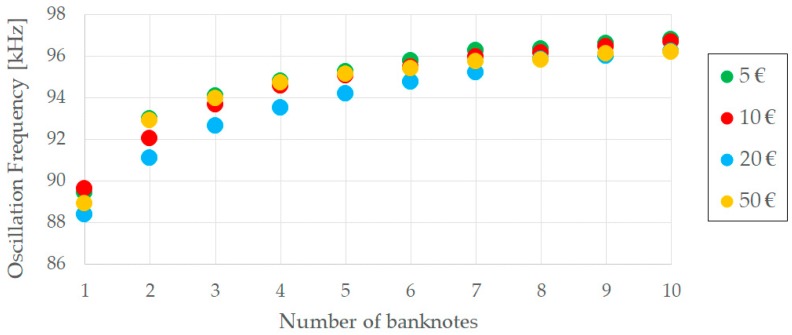
Evolution of the oscillation frequency according to the number of banknotes.

Since the capacitance of the parallel plate capacitor has been found to be in the order of picofarads (Figure 6), and aiming to obtain oscillation frequencies around tens of kHz, a 10 kΩ resistor has been chosen for *R_T_*. In this way, Figure 9 represents variations of the oscillation frequency as different banknotes are placed in between the parallel plate capacitor, thus proving the feasibility of the proposed solution.

This oscillation frequency is measured by a microcontroller that, using an internal counter module and an internal timer module, counts the number of cycles of the waveform generated per unit of time. The system described in this work comprises four oscillatory waveforms: one associated to each of the four banknote compartments considered (5€, 10€, 20€ and 50€). In order to minimize the resources required from the processing unit or microcontroller, a 4:1 multiplexer has been included in the electric circuit so that all the four waveforms can be multiplexed into the microcontroller, hence requiring only one counter module and one timer from the microcontroller. The multiplexer selected is ADG704 from Analog Devices, a CMOS analog multiplexer comprising four single channels [24]. Additionally, its single supply range and enable pin make it ideal for battery-powered instruments.

It must be clarified that the graph in Figure 9 shows the average frequency observed for each bank denomination and for different quantities. Actual data collected during the tests revealed that the frequency dispersion obtained remains within acceptable limits. For the same quantity of any given banknote denomination, several tests have been repeated to obtain the average frequency shown in Figure 9. In this way, the standard deviation obtained is always below 48 Hz. Since the frequencies produced by the system are around 90 kHz, this yields a measurement error of only 0.05%. Thus, it can be concluded that the current approach is capable of identifying different quantities of banknotes even if, because of their being worn out or wrinkled, they would show a slightly different change in their equivalent capacitance.

Furthermore, the differences in the frequencies generated for 6 banknotes with respect to the ones generated for 7 banknotes are still in the order of a few hundreds of hertz (the exact number varying depending on the banknote denomination) and the differences from 9 to 10 banknotes are still around 100 Hz. Given the measurement error indicated above, these frequency variations are suitable to easily identify these one-banknote increments.

At this point of the design, the data coming from the sensors are ready to be further processed. In the next Section, the integration of all the elements that make up the prototype is discussed.

## 5. System Integration

The proper functioning of the prototype proposed in this paper highly depends on the integration of all the different parts. So far, only the data acquisition system has been discussed. In this Section, the remaining elements needed to fully develop the prototype are included.

### 5.1. Central Processing Unit

All components mentioned so far are essential for both collecting and transmitting data, but, up to this point, these two actions are unconnected. There exists a need for a nexus between them, solved by means of a processing unit capable of receiving data through its input pins, processing them and generating other data that can either be stored or driven to any of its output pins.

Based on the little space available, the low-power requirement of the prototype and its low cost, a Microchip PIC24F32KA302 microcontroller has been chosen as the processing unit for the prototype. As well as some general I/O pins to control the position of the cash drawer and to control the external multiplexer indicated above, the microcontroller used must include the following internal modules: an analog-to-digital converter that receives the voltage representative of the coins’ weight; a counter and a timer to count the number of cycles of the waveform associated to the number of banknotes per unit of time; and a Universal Asynchronous Receiver/Transmitter (UART) that enables serial communication with a Bluetooth module in order to send the information to a portable device.

**Figure 10:**
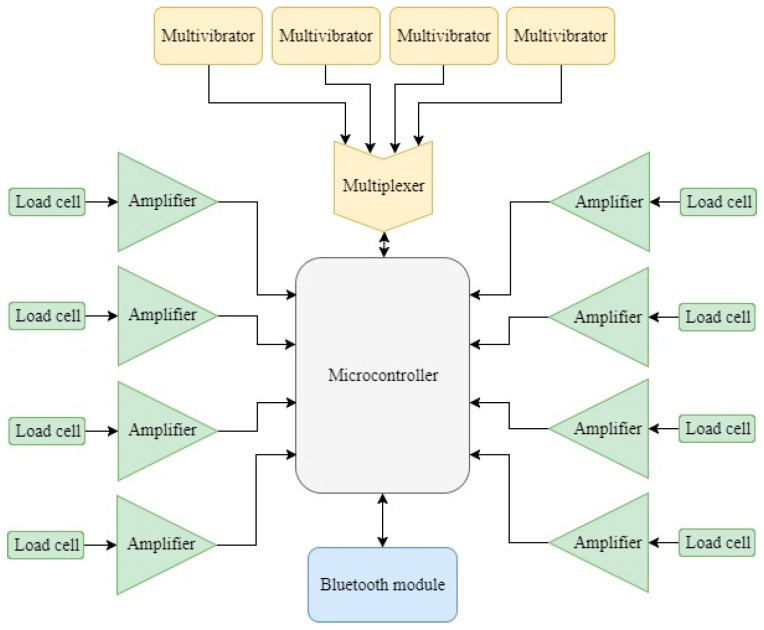
System overview.

An overview of the complete system is included in Figure 10.

In addition to the core functionalities of the prototype, additional features have been implemented so as to facilitate its utilization. Knowing that the status of the cash drawer (whether it is open or closed) simplifies the functioning of system to a large extent, measurements must only be made whenever the cash drawer is closed. In such a way, it has been decided to include a limit switch as an external interrupt source for the microcontroller. Every time the limit switch is activated, an event is triggered and the data acquisition of the sensors is started.

Another feature that has not been previously mentioned, but which is extremely common in today’s cash registers, is the possibility to automatically open the cash drawer whenever a transaction needs to be done. This feature has also been implemented in the prototype. The actuator that pushes the cash drawer out is an electromagnet driven by a general I/O of the microcontroller.

### 5.2. Communications

The microcontroller selected, which includes a serial communication module, makes it possible to transmit the sensor data as well as remotely interact with the prototype. In Section 2, the use of Bluetooth technology was already justified and based on those arguments; a commercial HC-05 Bluetooth Serial Port Protocol (SPP) module has been integrated into the system. By using this module, the implementation of Bluetooth communication is largely simplified, since it can be directly connected to any Universal Asynchronous Receiver/Transmitter (UART) module of the microcontroller. Thus, it is the HC-05 that is in charge of implementing all the Bluetooth communication protocol, making it transparent for the developer of the application and the programmer of the microcontroller unit, who will only have to decide the information to be shared.

In order not to allow the data to be accessed from any device in the surroundings, the communication has been password protected. The range of the communications is another factor that should be considered when designing the system. Several tests have been carried out with the prototype developed and the range oscillates from 15 m to 50 m, depending on the presence of obstacles.

### 5.3. Power Management

In the last years, Universal Serial Bus (USB) has evolved from a data interface capable of supplying limited power to a primary provider of power with a data interface. Thus, many small devices charge or get their power from USB ports [25]. Because of that, a Micro USB Type B connector has been included in the design of the prototype.

**Figure 11:**
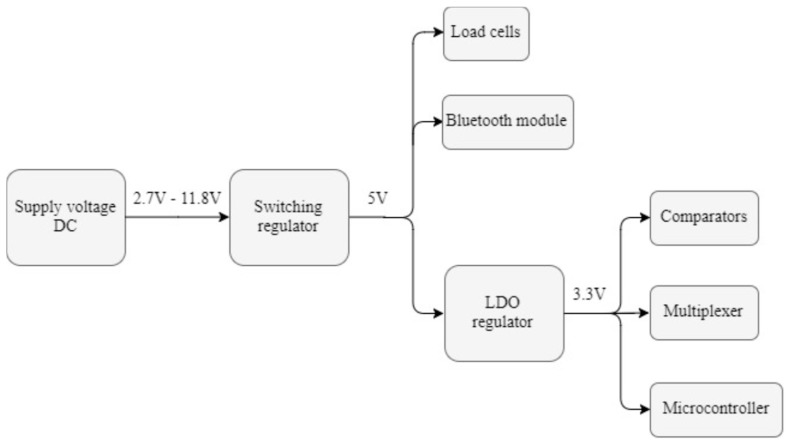
Structure of the power supply system.

According to the USB 3.1 Specification, the voltage supplied by any host (computer, external battery, …) can vary from 4.45 V to 5.25 V. Either way, the voltage provided by the host will be the supply voltage for the whole system [13]. However, not all the components previously selected have the same supply voltages and, therefore, a power management system is required. The overview of the system implemented in the prototype, which consists of first getting a 5-volt regulated voltage and 3.3 V afterwards, is shown in Figure 11.

Since the USB voltage can be either higher or lower than the desired 5 V, an S7V7F5 step-up/step-down regulator from Pololu has been used [26]. As indicated in Figure 11, this switching regulator can operate from input voltages between 2.7 V and 11.8 V, which extends the capabilities of the system designed: Not only can it operate when connected to a USB port, but also when supplied by many other DC sources, including batteries.

Once the regulated 5-volt voltage is available, different approaches can be followed to obtain 3.3 V out of it. Even though the efficiency of a switching regulator is higher than that of a Low Drop Out (LDO) regulator, the smaller size, lower quiescent current I_Q_, and easier implementation of the LDO turn it into the preferred option. Specifically, the regulator chosen is the TPS79733DC from Texas Instruments, a Low-Dropout Linear Regulator with ultra-low I_Q_ intended for use in microcontroller-based, battery-powered applications [27].

### 5.4. Mechanical Design

**Figure 12:**
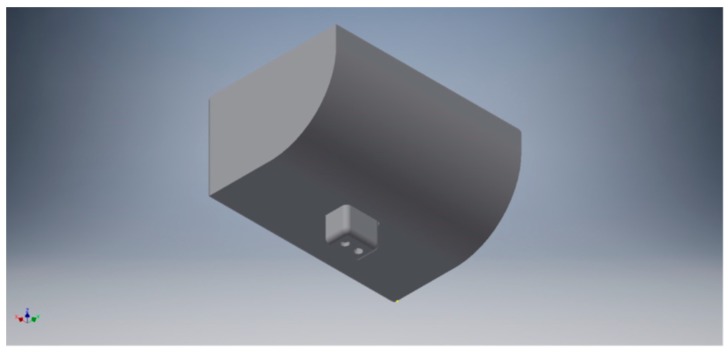
3D rendering of the assembly between a coin compartment and the workpiece designed for the union between compartments and load cells.

**Figure 13:**
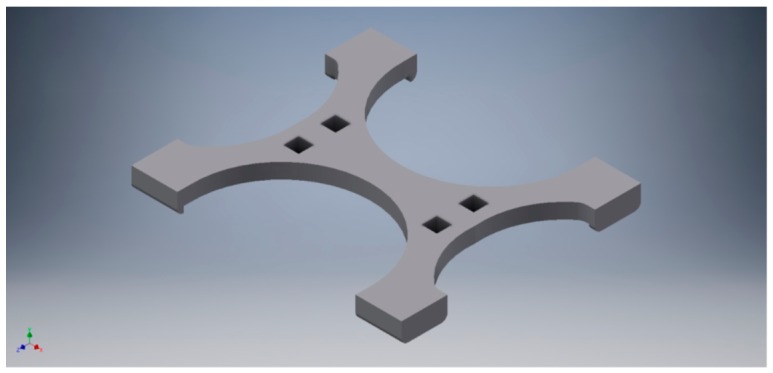
3D rendering of the workpiece the conductive plate of the parallel capacitor is glued to. This is an element of the new bill holder.

Cash registers available in the market today do not have a mechanical design that allows the implementation of the sensors required for the proper functioning of the prototype. Therefore, a commercial design of a cash drawer had to be modified, so that it could integrate all the instrumentation. All the mechanical parts of the prototype were manufactured using a Flashforge Creator Pro 3D printer [28] because of its speed, low cost and instant availability. Figure 12 shows the piece designed to attach one of the coin compartments with its corresponding load cell. In the same way, the mechanical part designed and manufactured to fix the top conductive plate of each of the capacitive sensor, is shown in Figure 13.

### 5.5. PCB Design

This Subsection covers the design of a Printed Circuit Board (PCB) that will integrate all different components, as well as the necessary connectors, in the most convenient way. In addition to that, possible constraints imposed by the mechanical design made in the previous section have also been considered. Based on these limitations, the next step consists in defining the arrangement of all different components and connectors in the most compact way over the PCB. This step, as well as the routing of all tracks, has been carried out using Altium Designer.

**Figure 14:**
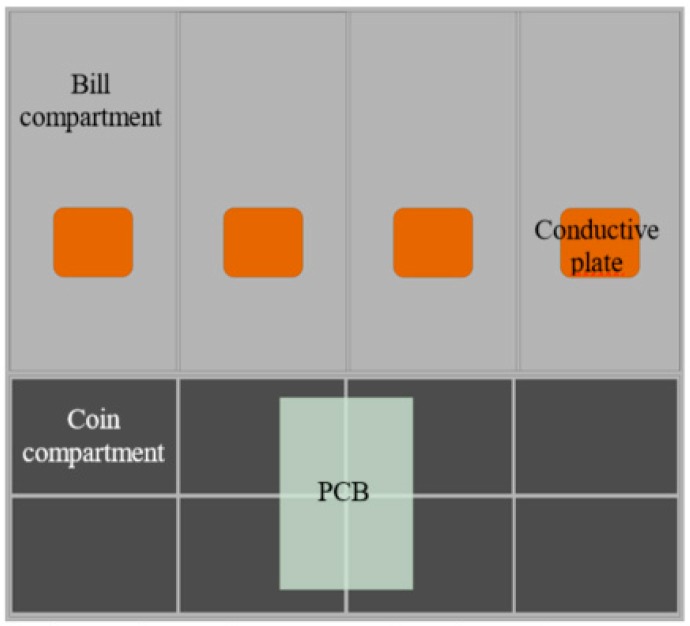
Cash drawer layout (350 mm × 300 mm) and PCB (60 mm × 100 mm) location.

The PCB is located under the coin compartments as shown in Figure 14. The headers meant to connect the load cells and the parallel plate capacitors to the PCB have been placed so as to minimize the distance between the sensors and the PCB, thus preventing the appearance of electromagnetic interference. Also, the PCB has been designed so that the two independent modules corresponding to the step-up/step-down converter and the Bluetooth communication can be accommodated within the available space of the PCB, thus resulting in a more compact design.

This completes the definition of the hardware of the system, which has been fully described. In the following Section, both the design and implementation of the software will be carried out.

## 6. Software Development

### 6.1. Microcontroller Program

In a previous Section, a microcontroller PIC24F32KA302 from Microchip was selected as the central processing unit of the system. In this Section, the design of the program that will allow this microcontroller to receive and process the data needed to fulfil the objectives of this project will be covered.

The program can be divided into two main parts: (a) initialization and configuration of all different modules of the microcontroller, and (b) definition of all the procedures undertaken during the execution of the main loop.

Assuming the cash drawer will be opened for inserting or withdrawing money, it can be stated that the amount of money deposited inside the cash drawer will only need to be measured once each time the cash drawer is closed. The opening action of the cash drawer can either be manual or automatic, thanks to the implementation of an electromagnet which is activated through Bluetooth from the user’s device. Hence, the program must be continuously checking for data received through the serial port. That is the only task performed in the main loop of the program, as all the other actions have been programmed to be handled by means of interrupt events. In the prototype developed, three events have been identified: opening the cash drawer, calibrating the system and updating the amount of money available in the cash register.

No matter how the cash drawer has been opened, whenever it is closed again a measurement must start. As soon as the cash drawer is closed, a limit switch will trigger an interrupt in the microcontroller that marks the beginning for the measurements.

**Figure 15:**
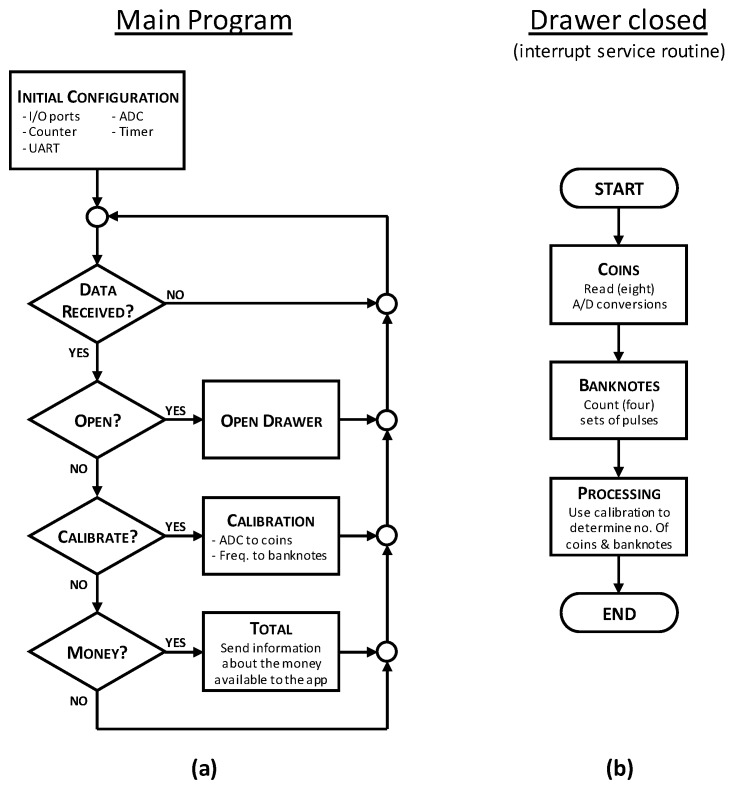
Flow diagram of (**a**) the main program of the microcontroller, and (**b**) the interrupt service routine associated to the cash drawer being closed.

In the interrupt service routine associated to the closing of the cash drawer, analog to digital conversions are carried out by means of the A/D module of the microcontroller and, at the same time, the oscillation frequency is being measured. Figure 15 shows a flow diagram illustrating the main tasks included in the main program and those associated to the execution of the interrupt service routine that is run when the cash drawer is closed.

According to Figure 15, as soon as all the conversions have finished, the data obtained are immediately processed. The data processing part of the program includes the transformation of the measurements taken from the real world into integer numbers, which represent the quantities of all the different coins and banknotes denominations present in the cash drawer. At this point, the need for calibration curves becomes evident. These curves play a key role in the proper functioning of the system, as they define the final results. Because of this, the calibration parameters that allow measurements to be translated into number of coins and banknotes have been saved in a non-volatile memory, such as the EEPROM included in the microcontroller.

**Figure 16:**
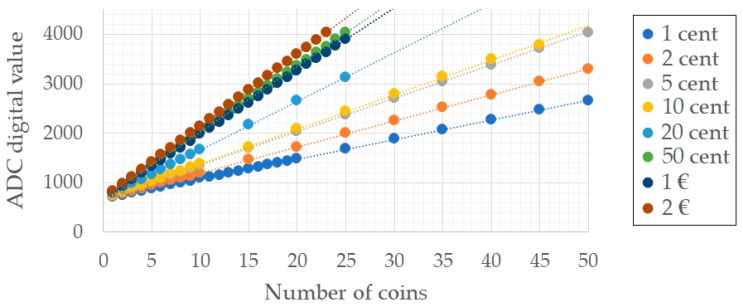
Graphical representation of the analog-to-digital conversion performed by the AD module (12-bit resolution).

Figure 16 shows that the result of the A/D conversions is directly proportional to the number of coins and, therefore, the only parameters to obtain for the calibration curve are the slope and the intersection point with the Y-axis of the plot, which relates the number of coins and the ADC results. Assuming a linear performance of the load cells over time, the values of the slopes will remain constant. However, it is true that any variation in the weight of the empty compartment can cause an offset error. Therefore, the calibration process for the coins consists in the adjustment of the empty compartment ADC values.

The calibration curve used for the banknotes has not been obtained as directly as the one associated to coins, the main reason being that the oscillation frequency does not evolve linearly with the number of banknotes (see Figure 9). Because of that, a single-point or even a two-point calibration will not result in proper accuracy of the calibration curve. This is why a multiple-point calibration has been performed instead, with linear interpolation being used to calculate the intermediate points.

### 6.2. App User Interface

In the previous Section, the interaction of the user through a Bluetooth-connected device has been mentioned. Controlling the prototype from a hand-held device, such as a smartphone or a tablet, is also one of the objectives of the project. Therefore, an application has been developed that will act as an interface between the prototype and the user.

According to a statistical study conducted by the International Data Corporation (IDC), Android dominates the smartphone market with a share of 85% [29]. Because of that, along with the huge amount of information and resources available, Android operating system has been chosen as the preferred alternative. Among all different tools intended for the development of Android applications, MIT App Inventor was used, a development software environment created by Massachusetts Institute of Technology (MIT) [30].

The main idea is to use the app as a graphical interface, so that the user can see the amount of money inside the cash drawer in real-time. In addition to that, the application must allow the user to open the cash drawer and to perform a complete calibration of the prototype at any time, as already anticipated in the previous Section of this paper.

**Figure 17:**
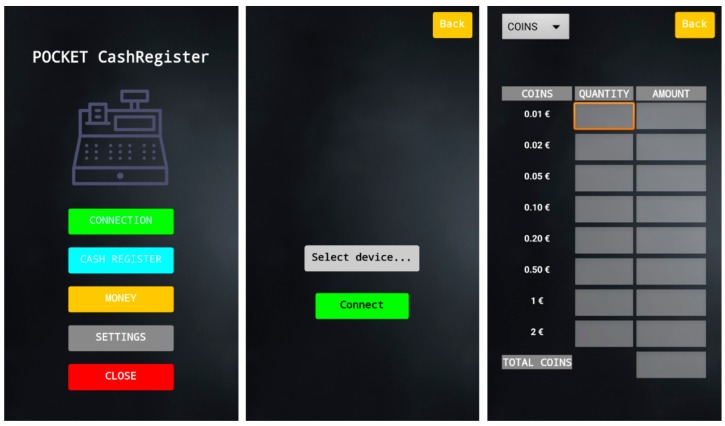
Screenshots of the Android application. From left to right, Main menu of the application, CONNECT screen, and MONEY screen.

As it can be observed from the main menu shown in Figure 17, the user can act over five different buttons. The top button will open a new screen that allows users to select and connect the smartphone to any Bluetooth device detected within its Bluetooth range.

The second button of the main menu leads to another screen that allows the user to open the cash drawer remotely.

It is the screen accessed through the MONEY button that shows the amount of money inside the cash drawer. A dropdown menu on the top left corner of the screen gives the user the chance to show either the amount of coins, banknotes, or the totals.

A calibration of the prototype can be started by entering the SETTINGS menu and clicking the calibration button. The cash drawer will open if it was closed and several messages will pop up on the screen, guiding the user through the whole calibration process.

Finally, as its name suggests, the CLOSE button will end the application.

## 7. Experimental Validation

Throughout the development of the complete system, the performance of different elements has been tested, and some of the most relevant results are summarized in this Section.

The feasibility of the coins counting solution proposed in this paper has been proven with several tests, whose results have been presented in Figure 15. As it can be seen, there exists a linear relationship between the number of coins and the AD converter value, which makes it possible to directly determine the number of coins of each denomination. Error detection techniques could also be implemented based on the results obtained.

As for the banknote counting solution, it should be noted that the capacitance varies according to the number of banknotes (see Figure 6), as expected. At least three different measurements were made for each banknote denomination. These measurements used different quantities of banknotes (from 1 to 10) and with different qualities too (new, worn, wrinkled). The tests performed revealed good accuracy and repeatability of the frequencies representing the quantity of banknotes for each denomination. Table 3 illustrates this by showing the average value and the standard deviation obtained for the different denominations and for quantities up to 10 units. The low values obtained for the standard deviation prove the goodness of the method defined.

It has been observed during the tests that placing a banknote in different positions with respect to the parallel plates produces small variations in the capacitance, thus introducing a limitation to the solution proposed. Because of that, all the experiments have been performed by keeping the relative position between the parallel plates of the capacitor and the banknotes constant. This constraint should not pose any limitation to the application of the measurement method described in this work, since the four compartments meant to store the banknotes will contribute to placing them in the appropriate position.

Another conclusion that can be derived from the data collected by the sensors is that the size of the different banknote denominations has an effect over the capacitance. Although this fact has not been taken into consideration in the prototype developed, it could be used to identify different banknote denominations placed in the same compartment.

Additional tests have been performed in order to check other functionalities of the system. For instance, as mentioned in Section 5.2, the range of the Bluetooth module has been tested. This was done by transferring a known number of data packages between a secondary device and the prototype. Up to 50 m away (without obstacles), all the packages sent were correctly received at their destination; this determined the maximum distance the system will be reliable at.

Since the prototype has also been designed with the capability to be a portable device, its average consumption was also measured. The measurement was performed with all the peripherals enabled, and the average current was found to be less than 100 mA.

## 8. Conclusions

In this project, a cash register prototype capable of automatically counting the amount of money deposited inside has been developed. Each time the cash drawer is closed, a measurement procedure starts. The development of the prototype may be split into two different subsystems, one in charge of counting the number of different coins, and the other in charge of counting the number of banknotes.

The solution proposed for counting the coins is based on the working principle of a scale. By means of load cells, each coin denomination is weighted individually using a different coin compartment, thus allowing error detection in the introduction or withdrawal of money as well as simplifying the software implementation.

With regard to the banknotes, an innovative solution based on capacitance measurement has been developed. No record or precedent of an implementation of a banknote-counting sensor like the one presented in this paper has been found. Capacitive measurement of distances is a well-known technique but, to the knowledge of the authors of this work, has not been used before to determine the number of banknotes in a compartment. The tests performed have determined that the implementation of the sensor carried out allows identifying at least 10 banknotes of each denomination. Currently, its usage leads to a limitation associated to the way the banknote is deposited in the compartment. Nonetheless, further testing and development of the solution may overcome this constraint.

This solution is easily adapted to currencies from different countries by simply including in the microcontroller program the data that relates weight with number of coins and frequency with number of banknotes. This is done by means of parameters and lookup tables that will be stored in the non-volatile memory of the microcontroller. Although this could be accomplished through the Android application developed, it is preferable to include this information in the microcontroller firmware for the sake of security.

Special attention should be paid to the Bluetooth communication link established between the cash register prototype and an Android device by means of a simple application. Although in this project it has been chosen to develop an Android application, the idea may be extrapolated to any other platform. In its current version, the prototype is meant to operate locally only, using the Android device as a simple graphical interface. However, it is possible to extend its functionality by including a Wi-Fi module (instead of the Bluetooth one) and developing a cloud application.

The bill of materials for the whole prototype costs around 230€. This price includes the cost of an HS-140 Seypos cash drawer that must be modified so as to include the capacitive sensors. Also, this price does not take into account the possible price reduction that could be obtained when buying large quantities.

The aspects previously mentioned set the fundamental pillars of the cash register prototype and the results obtained during testing show the feasibility of the solution proposed. In any case, further research may result in the improvement of the system performance.

## Figures and Tables

**Table 1:** Features of the Euro Coins.

Value (€)	Diameter (mm)	Thickness (mm)	Weight (g)
0.01	16.25	1.67	2.30
0.02	18.75	1.67	3.06
0.05	21.25	1.67	3.92
0.10	19.75	1.93	4.10
0.20	22.25	2.14	5.74
0.50	24.25	2.38	7.80
1	23.25	2.33	7.50
2	25.75	2.20	8.50

**Table 2:** Features of the Euro Banknotes.

Value (€)	Size (mm)	Colour
5	120 × 62	Grey
10	127 × 67	Red
20	133 × 72	Blue
50	140 × 77	Orange
100	147 × 82	Green
200	153 × 82	Yellow-brown
500	160 × 82	Purple

**Table 3:** Frequencies measured for each banknote denomination.

No. of Units	5€		10€		20€		50€	
	µ (Hz)	σ (%)	µ (Hz)	σ (%)	µ (Hz)	σ (%)	µ (Hz)	σ (%)
1	89,432	0.041	89608	0.015	88,372	0.030	88,902	0.055
2	92,990	0.007	92,041	0.017	91,084	0.005	92,925	0.022
3	94,090	0.013	93,654	0.007	92,664	0.022	93,966	0.017
4	94,782	0.031	94,584	0.012	93,533	0.013	94,738	0.030
5	95,246	0.028	95,079	0.008	94,207	0.015	95,142	0.002
6	95,791	0.006	95,472	0.003	94,750	0.021	95,396	0.012
7	96,253	0.007	95,969	0.002	95,228	0.010	95,740	0.018
8	96,331	0.010	96,141	0.011	95,856	0.013	95,806	0.013
9	96,605	0.008	96,478	0.007	96,013	0.024	96,110	0.021
10	96,783	0.014	96,668	0.004	96,219	0.016	96,185	0.025
